# Phosphoinositide-dependent protein kinase-1 (PDK1)-independent activation of the protein kinase C substrate, protein kinase D

**DOI:** 10.1016/j.febslet.2007.06.060

**Published:** 2007-07-24

**Authors:** C. David Wood, April P. Kelly, Sharon A. Matthews, Doreen A. Cantrell

**Affiliations:** Division of Cell Biology and Immunology, College of Life Sciences, MSI/WTB/CIR Complex, University of Dundee, Dow Street, Dundee DD1 5EH, United Kingdom

**Keywords:** ES cell, embryonic stem cell, p90RSK, p90 ribosomal S6 kinase, PDK1, 3′-phosphoinositide-dependent protein kinase-1, PKC, protein kinase C, PKD, protein kinase D, S6K1, 70-kilodalton ribosomal S6 kinase 1, PDK1, PKD, PKC

## Abstract

Phosphoinoisitide dependent kinase l (PDK1) is proposed to phosphorylate a key threonine residue within the catalytic domain of the protein kinase C (PKC) superfamily that controls the stability and catalytic competence of these kinases. Hence, in PDK1-null embryonic stem cells intracellular levels of PKCα, PKCβ1, PKCγ, and PKC*ε* are strikingly reduced. Although PDK1-null cells have reduced endogenous PKC levels they are not completely devoid of PKCs and the integrity of downstream PKC effector pathways in the absence of PDK1 has not been determined. In the present report, the PDK1 requirement for controlling the phosphorylation and activity of a well characterised substrate for PKCs, the serine kinase protein kinase D, has been examined. The data show that in embryonic stem cells and thymocytes loss of PDK1 does not prevent PKC-mediated phosphorylation and activation of protein kinase D. These results reveal that loss of PDK1 does not functionally inactivate all PKC-mediated signal transduction.

## 1 Introduction

Phosphoinoisitide dependent kinase l (PDK1) phosphorylates a key threonine residue within the catalytic domain of a number of AGC family kinases, including diacylglycerol-regulated kinases of the protein kinase C (PKC) superfamily and phosphatidyl inositol-3 kinase (PI3K)-controlled serine kinases such as Akt/PKB and the 70-kilodalton ribosomal S6 kinase 1 (S6K1) [1]. PDK1-mediated phosphorylation of many of these AGC kinases is essential for their activation. Accordingly, deletion of PDK1 by homologous recombination removes one of the rate-limiting regulators of multiple serine kinases and causes embryonic lethality [2]. To circumvent this prenatal death and explore the role of PDK1 in different tissues, mice expressing PDK1 alleles flanked with the loxP Cre excision sequence (PDK1^flΔneo/flΔneo^ mice) have been generated. These were first used to achieve muscle specific deletion of PDK1, producing mice that die early with severe defects in cardiac function [3]. The importance of PDK1 for T lymphocyte biology has also been explored by looking at T cell development following T lineage specific deletion of PDK1. These studies showed that deletion of PDK1 in T cell progenitors in the thymus results in a block in T cell differentiation, indicating that PDK1 has essential functions in T lymphocyte development [4].

The importance of PDK1 for T cell development in the thymus focuses attention on the identification of the PDK1 substrates directly involved in mediating the unique and non-redundant functions of this kinase in vivo. In this context, studies of mouse embryonic stem cells in which both PDK1 alleles have been deleted have shown that PDK1 is necessary for T-loop phosphorylation, and catalytic activity, of the AGC kinases Akt/PKB, S6K1 and p90 ribosomal S6 kinase (p90RSK) [5]. However, PDK1 is not required for the phosphorylation and activation of other AGC kinases such as PKA or AMPK [5].

One very important subfamily of AGC kinases that can be regulated by PDK1 are the protein kinase C enzymes. There are multiple isoforms of PKC: the classical PKCs (α, βI, βII and γ) which are regulated by calcium, diacylglycerol (DAG) and phospholipids; novel PKCs (δ, ε, η and θ) which are regulated by DAG and phospholipids; and the atypical PKCs (ζ and λ) which are insensitive to both calcium and DAG. The catalytic domain of PKCs have a conserved T-loop motif that is a substrate for PDK1, and it has been proposed that PDK1 is responsible for T-loop phosphorylation of the various PKC isoforms [6–8]. PDK1-mediated T-loop phosphorylation of PKCs is proposed to stabilize protein expression by acting as a priming signal that enables PKCs to achieve catalytic maturity [6,7]. Here, PDK1 docks to a hydrophobic motif in the C-terminus of newly synthesised PKC enzymes, enabling it to phosphorylate their T-loop sites. Following T-loop phosphorylation, PDK1 disengages enabling PKCs to autophosphorylate their C-terminal hydrophobic motifs and achieve catalytic maturity [9–11]. Once mature, PKCs remain inactive due to autoinhibition from their pseudosubstrate motifs and are only activated following the generation of second messengers, such as diacylglycerol and calcium.

The release of PDK1 from PKCs is rate limiting and so PDK1 is responsible for controlling the amount of mature/catalytically competent PKCs expressed in cells [11]. Hence, in the absence of PDK1, intracellular levels of PKCα, PKCβ1, PKCγ, and PKCε are strikingly reduced [6,12]. PKCξ levels are similar in wild-type and PDK1-null embryonic stem cell (ES cells) although PKCξ is not phosphorylated on its T-loop Thr410 site in the absence of PDK1 [6,13]. PKCδ expression is also reduced in PDK1-null cells [6,12] although there was still phosphorylation of the PKC*δ* T-loop site Thr505 in the residual pool of PKCδ still present in PDK1-null cells [13]. Moreover, there is some evidence in the literature that T-loop phosphorylation is not absolutely critical for PKCδ catalytic activity, since a bacterially-expressed Thr505A PKCδ mutant protein retains some activity in vitro [14]. Of note, a glutamic acid residue at position 500 within the PKCδ activation loop, which is important for catalytic activity [15], could potentially compensate for the lack of PKCδ T-loop phosphorylation in PDK1-null cells.

These studies support the hypothesis that PDK1 has an important role in regulating PKC phosphorylation and stability. However, it has not been shown unequivocally that the reduced levels of PKCs in PDK1-null cells abrogate PKC-mediated cellular responses. In this context, it has been shown that in the absence of PDK1 there is loss of Akt/PKB and S6K1 T-loop phosphorylation and a corresponding inability of cells to regulate phosphorylation of Akt/PKB or S6K1 substrates [5]. There is thus confidence that Akt/PKB and S6K1 function is defective in PDK1-null cells. This has not been determined for the PKCs because although PDK1-null cells have reduced endogenous PKC levels the integrity of downstream PKC effector pathways in PDK1-null cells has not been determined.

One well characterised target for novel PKCs is the serine kinase protein kinase D (PKD). Activation of PKD requires the phosphorylation of two key serine residues within the catalytic domain [16,17]. These residues are absolutely conserved through evolution from *C. elegans* to man and are substrates for novel PKCs. The activation of PKCs is thus required for PKD activation in a variety of cell lineages in response to a number of different stimuli [16,17]. For example, it has been demonstrated that PKD activity in lymphocytes is regulated by PKC-mediated phosphorylation of its catalytic domain [18,19] and phosphorylation of the PKD catalytic domain is reduced in PKCβ-deficient spleenocytes [20]. It would therefore be expected that PKD would remain inactive if PKCs cannot achieve catalytic maturity in PDK1-null cells. In support of this hypothesis, previous work has established that co-expression of PDK1 and the novel PKCε isoform, together with PKD, enhances PKD activity [21]. The hypothesis that PKD activation is dependent on PDK1 has not been fully investigated and is important to address as it is one way to assess whether there is residual functional PKC activity in PDK1-null cells. Accordingly, the objective of the present study was to examine whether loss of PDK1 results in loss of the PKC signalling pathway that regulates PKD.

## 2 Results and discussion

### 2.1 Activation of protein kinase D in PDK1^−/−^ thymocytes

**Fig. 1:**
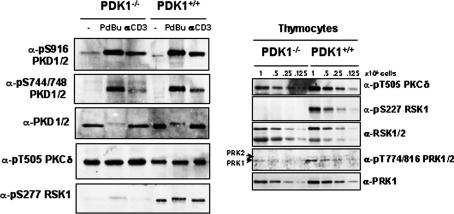
PKD activity in PKD1-null pre-T cells. DN4 thymocytes, purified from wild-type (PDK1^+/+^) and LckCre^+^PKD1^flΔneo/flΔneo^ (PKD1^−/−^) mice, were left unstimulated (−) or were treated with PdBu (20 ng/ml) for 10 min before lysis. Protein extracts from 1 × 10^6^ cells (unless otherwise indicated) were separated by SDS–PAGE and analyzed by Western blotting with the indicated antibodies. PKD activity was assessed using a phospho-specific antibody against its Ser916 autophosphorylation site.

To investigate the impact of PDK1 loss on protein kinase D activation in PDK1 deficient T lymphocytes, mice expressing PDK1 alleles flanked with the loxP Cre excision sequence (PDK1^flΔneo/flΔneo^) were bred with transgenic mice expressing Cre recombinase under the control of the proximal p56lck promoter which induces expression of Cre in T cell progenitors in the thymus. It has been shown previously that in LckCre^+^PDK1^flΔneo/flΔneo^ mice PDK1 is deleted in pre-T cells, preventing normal T cell development [4]. To explore the role of PDK1 in the regulation of PKD activity, wild-type or PDK1^−/−^ pre-T cells were isolated and left unstimulated, stimulated with a crosslinking α-CD3 antibody (to activate preTCR signalling) or were stimulated with the phorbol ester PdBu (a diacylglycerol mimetic which activates classical/novel PKCs), as indicated. PKD catalytic activity was monitored using an antisera that recognises PKD molecules that are autophosphorylated on Ser916 [22]. The data (Fig. 1) show that in quiescent wild-type thymocytes PKD is not autophosphorylated on Ser916 whereas α-CD3 or PdBu stimulation strongly induced PKD catalytic activity, as shown by increased Ser916 autophosphorylation. The results also show that both α-CD3 and PdBu treatment induce strong PKD Ser916 autophosphorylation in PDK1^−/−^ pre-T cells (Fig. 1). PKD activation is dependent on PKC-mediated phosphorylation of two key serine residues (Ser744/S748) within the PKD catalytic domain and as shown in Fig. 1, PKD is phosphorylated on Ser744/S748 following *α*-CD3 or PdBu activation in both wild-type and PDK1^−/−^ pre-T cells.

The phosphorylation and activation of PKD in PDK1^−/−^ pre-T cells argues that these cells still contain a functional, catalytically competent PKC(s) that is able to respond to diacylglycerol/phorbol ester signals. RNAi experiments have demonstrated that PKCε in Swiss 3T3 cells or PKCδ in HeLa cells are the PKC isoforms responsible for serine phosphorylation (Ser744/S748) within the PKD activation loop [23,24]. PKCδ has been shown to be residually phosphorylated on its T-loop Thr505 site in PDK1-null ES cells [13]. We therefore examined the phosphorylation status of the PKCδ T-loop site, Thr505, in PDK1^−/−^ pre-T cells. The data in Fig. 1 show basal phosphorylation of PKCδ Thr505 in wild-type pre-T cells and also show that significant phosphorylation of PKCδ on Thr505 in retained in PDK1^−/−^ pre-T cells. In contrast, as previously demonstrated, PDK1^−/−^ pre-T cells have lost activity of the PKB/S6K1 signalling pathway that controls the phosphorylation of the ribosomal S6 subunit on Ser235/236 [4,25]. The data in Fig. 1 also explore the impact of PDK1 loss on the T-loop phosphorylation of p90RSK1 and PRK1. The data show Western blot analysis of wild type and PDK1 null pre-T cells with a phospho-specific antibody against Ser227 in p90RSK1 and show that this site is basally phosphorylated in wild-type pre-T cells but is not phosphorylated in PDK1-null pre-T cells. Similarly, basal phosphorylation of PRK1 on its Thr774 T-loop site is substantially reduced in PDK1-null pre-T cells. Thus, PDK1-null pre-T cells show defective activation of some, but not all, AGC family kinases: regulation of a PKC–PKD signalling pathway remains intact in these cells.

### 2.2 Activation of protein kinase D in PDK1^−/−^ ES cells

**Fig. 2:**
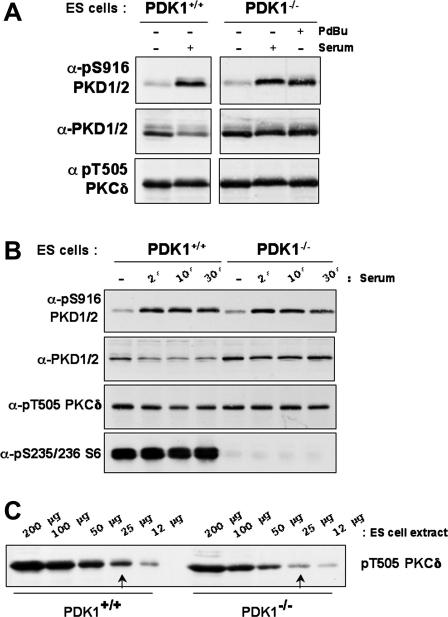
PKD activity in PKD1-null embryonic stem cells. (A) Serum-starved wild-type (PKD1^+/+^) and PKD1^−/−^ murine ES cells were left unstimulated (−) or were treated with PdBu (20 ng/ml) or serum (10%) for 10 min before lysis. Protein extracts were separated by SDS–PAGE and analyzed by Western blotting with the indicated antibodies. (B) Serum starved wild-type and PKD1^−/−^ murine ES cells were left unstimulated (−) or were treated with/without serum (10%) for varying times before lysis. Protein extracts (200 μg) were separated by SDS–PAGE and analyzed by Western blotting with the indicated antibodies. (C) Protein extracts (200–12 μg) from wild-type and PKD1^−/−^ murine ES cells were separated by SDS–PAGE and analyzed by Western blotting with a pThr505 PKCδ antibody.

**Fig. 3:**
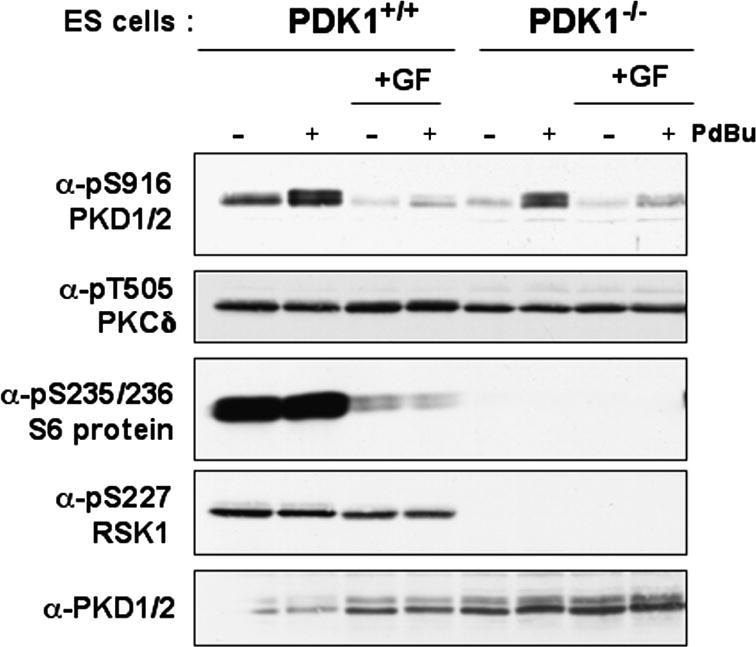
PKD activity in PKD1-null ES cells is sensitive to the PKC inhibitor, GF109203X. Wild-type and PKD1^−/−^ murine ES cells were pretreated with 5 μM GF109203X (GF) for 20 min, then left unstimulated (−) or were treated with PdBu (20 ng/ml) for 10 min before lysis. Protein extracts (200 μg) were separated by SDS–PAGE and analyzed by Western blotting with the indicated antibodies.

The experiments in Fig. 1 show that PDK1 is not essential for PKD activation in thymocytes. To probe further the involvement of PDK1 in the regulation of PKC–PKD signalling, experiments in wild-type and PDK1^−/−^ mouse embryonic stem cells were performed. Western blot analysis of wild-type and PDK1^−/−^ ES cells with a PKCδ Thr505 phosphospecific antisera confirm that PKCδ Thr505 phosphorylation is detectable, albeit slightly reduced, in PDK1-null versus wild-type ES cells (Fig. 2A–C). To probe PKD activity, cells were left unstimulated or activated by exposure to serum or phorbol ester as indicated and PKD catalytic activity monitored using the pSer916 antisera. In quiescent, wild-type ES cells, PKD was not autophosphorylated on Ser916 but both serum and PdBu stimulation strongly and rapidly induced PKD activity as measured by increased Ser916 autophosphorylation (Fig. 2A and B). Serum or PdBu stimulation also induced PKD activity in the PDK1^−/−^ ES cells (Fig. 2A and B) whereas p90RSK1 T-loop phosphorylation (Ser227) was basally detectable in wild-type ES cells but not in the PDK1^−/−^ ES cells (Fig. 3). S6K1 signalling was also abrogated, as expected, in the PDK1^−/−^ ES cells, as shown by the lack of phosphorylation of its substrate, the S6 ribosomal protein subunit (Fig. 2B).

The ability of serum and phorbol esters to activate PKD in PDK1-null ES cells is an indication that there is residual, catalytically competent PKC in these cells. PKC-mediated activation of PKD can be prevented by the PKC inhibitor GF109203X [17]. Therefore, the effects of GF109203X, on PKD activation in wild-type or PDK1^−/−^ ES cells was examined. The data (Fig. 3) show that PKD was phosphorylated on Ser916 in activated PDK1^+/+^ and PDK1^−/−^ cells but that PKD autophosphorylation on Ser916 was prevented by prior treatment of both cell types with GF109203X.

## 3 Concluding remarks

Loss of PDK1 in ES cells causes a reduction in levels of PKCα, β_I_, γ, δ, and ε [6,12] but it was not resolved whether the residual PKC in these cells is functional. As discussed above, there is a large body of evidence that PKD activation is mediated by novel PKCs such as PKCδ and ε and it has previously been proposed that PDK1 is required for activation of PKD [21], reflecting the requirement of PDK1 for phosphorylation of the key priming T-loop phosphorylation sites in PKCs. However, the present study shows that the hypothesis that PKD activation is dependent on PDK1 is incorrect. The reason this model fails is that there is not a complete loss of PKC activity in the absence of PDK1. We show that in PDK1-null pre-T cells PKCδ is still phosphorylated on its T-loop site Thr505; hence activation of pre-T cells with phorbol esters can still induce phosphorylation of PKD on its PKC substrate sequences Ser744/S748, inducing PKD activation and subsequent autophosphorylation on Ser916. In addition, the present data show that PKD can also be activated by phorbol esters or serum stimulation of PDK1-null ES cells in a PKC-regulated manner. Thus, the present results showing activation of PKD in PDK1^−/−^ cells is evidence that at least one PKC-mediated signalling pathway is intact in the absence of PDK1.

While loss of PDK1 does not prevent the activation of PKD, the issue of whether the efficiency of PKD activation is affected in PDK1-null cells requires further experimentation. While robust activation of PKD signalling is observed in PDK1-null ES cells and pre-T cells in response to phorbol esters or CD3 crosslinking we cannot exclude the possibility that PKD activation may be compromised in PDK1-null mature peripheral T cells during physiological peptide:MHC stimulation, however these experiments await the development of genetic models to test this.

In summary, PDK1 is essential for pre-T cell development and its loss is associated with loss of PKB/S6K1-mediated signal transduction [4,25]. The present results now show loss of p90RSK and PRK1 T-loop phosphorylation in PDK1^−/−^ pre-T cells but reveal that the PKCδ T-loop site Thr505 is still phosphorylated in PDK1-null pre-T cells. It has been proposed that the Thr505 T-loop site in PKCδ (as well as the corresponding T-loop site in PKCθ, [26]) can be autophosphorylated, which may explain why PKCδ Thr505 is still phosphorylated in the PDK1-null cells. Alternatively the present results could argue for the existence of another PKCδ priming kinase in developing thymocytes. Whatever the molecular basis for the continued phosphorylation of PKCδ following the loss of PDK1, the present results show that there is still residual functional PKC activity once PDK1 is lost. Hence, the importance of PDK1 for T cell development must reflect the requirement for PDK1 mediated phosphorylation of other members of the AGC serine kinase family, such as Akt/PKB, S6K1 or p90RSK, since phosphorylation and activity of these kinases is strictly dependent on PDK1 expression.

## 4 Materials and methods

### 4.1 Cell preparation and stimulation

PDK1^+/+^ and PDK1^−/−^ murine embryonic stem cells [5] were grown on gelatinised tissue culture plates in Knockout DMEM supplemented with 10% knockout serum replacement, 15% foetal bovine serum (Hyclone), 0.1 mM non-essential amino acids, antibiotics (100 U/ml penicillin G, 100 μg/ml streptomycin and 1 μg/ml ciprofloxacin [Bayer Pharmaceuticals]), 2 mM l-glutamine, 1 mM sodium pyruvate, 0.1 mM β-mercaptoethanol and 25 ng/ml murine leukaemia inhibitory factor (LIF).

### 4.2 Mice

All mice used were between 5 and 7 weeks of age. Mice were bred and maintained in specific pathogen-free conditions and animal experimentation was approved by Home Office project license PPL60/3116. T-PDK1^−/−^ mice were generated by crossing mice with PDK1 floxed alleles (PDK1^flΔneo/flΔneo^) with mice expressing *Cre* recombinase under the control of the p56^*lck*^ promoter to ablate expression of PDK1 in T cells [2,4]. Control mice used for analyses of T-PDK1^−/−^ mice were age-matched phenotypically normal littermates.

### 4.3 Purification of thymocyte subpopulations

Antibodies conjugated to fluorescein isothiocyanate (FITC), phycoerythrin (PE), allophycocyanin (APC) and biotin were obtained from PharMingen. Cells were stained for surface expression of the following markers: CD4, CD8, CD25, CD44, CD3ε, γδ, B220, and Thy1. The DN4 subpopulation were defined by their cell surface marker expression and subsequently sorted using a Vantage cell sorter (Becton Dickinson) or AutoMacs magnetic cell sorter (Miltenyi Biotec).

### 4.4 Cell lysis and Western blot analysis

Cells were lysed for 10 min at 4 °C (20 × 10^6^ cells/ml) using buffer: 100 mM HEPES, pH 7.4, 150 mM NaCl, 1% NP40, 20 mM sodium fluoride, 20 mM iodoacetamide, 2 μM EDTA, 1 mM sodium orthovanadate, 2 μg/ml pepstatin A, 2 μg/ml leupeptin, 2 μg/ml chymostatin, 2 μg/ml antipain, 40 mM β-glycerophosphate and 1 mM phenylmethylsulfonylflouride. Soluble proteins were concentrated by precipitation with 1.5 vol. of acetone and separated by 8% sodium dodecyl sulfate–polyacrylamide gel electrophoresis (SDS–PAGE), transferred to polyvinylidene difluoride membranes and detected by Western blot analysis with the indicated antibodies (Cell Signalling Technology).
